# Calculation Simulation of Equivalent Thermomechanical Properties of Dispersion Nuclear Fuel

**DOI:** 10.3390/ma18235266

**Published:** 2025-11-21

**Authors:** Haoqi Yu, Tenglong Cong, Jie Zhang

**Affiliations:** 1 State Key Laboratory of Nuclear Power Safety Technology and Equipment, Shanghai Jiao Tong University, Shanghai 200240, China; 2 School of Nuclear Science and Engineering, Shanghai Jiao Tong University, Shanghai 200240, China; 3 Institute of Geotechnical Engineering, Zhejiang University, Hangzhou 310058, China

**Keywords:** equivalent properties, dispersed nuclear fuel, finite element method

## Abstract

The equivalent performance parameters of dispersion fuels are critical indicators for reactor safety analysis and fuel element evaluation. This study develops a numerical method to simulate the thermomechanical coupling behavior of metal matrix dispersion fuel rods at the mesoscopic scale and to calculate their macroscopic equivalent properties. Based on a fission gas migration model and considering irradiation effects, a thermomechanical–fission gas migration coupling method is established for metal matrix dispersion fuels. The effects of particle volume fraction, particle size, temperature, and burnup on the equivalent performance parameters are systematically analyzed and fitting formulas for the equivalent properties are provided. The results show the following: (1) The equivalent elastic modulus and shear modulus increase with particle volume fraction but decrease with temperature, and they exhibit a decreasing-then-increasing trend with burnup. (2) The equivalent thermal expansion coefficient increases with both particle volume fraction and temperature, while particle size has little effect. This study provides a theoretical basis for the optimization of dispersion fuel design and contributes to enhancing reactor core safety.

## 1. Introduction

**Figure 1:**
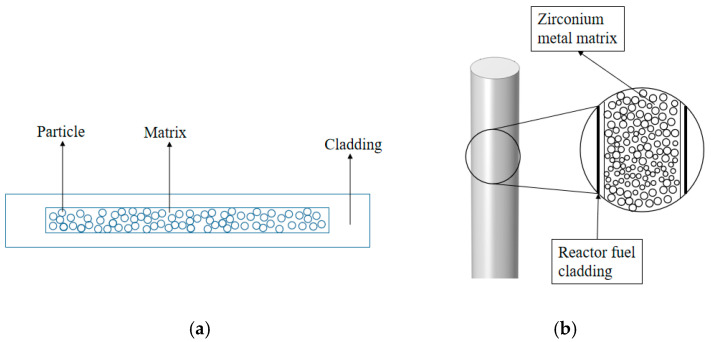
Schematic diagram of the fuel element. (**a**) Plate-type dispersion fuel element; (**b**) rod-type dispersion fuel element.

Nuclear fuel consists of materials that liberate energy through nuclear reactions inside a reactor. Depending on the reactor concept, fuels are typically classified as ceramic, metallic, liquid, or dispersion types [1]. Particle dispersion fuel represents a newer high-performance option in which fissile material is embedded as fine particles within a non-fissile matrix. By geometry, dispersion elements are further organized into plate-type and rod-type configurations [2], as shown in Figure 1. Notably, dispersion fuels generally display better irradiation stability than metallic fuels. Compared with ceramics, they offer higher thermal conductivity and a lower tendency toward cracking or catastrophic fracture. Compared with liquid fuels, they lend themselves to a wider set of operating scenarios. Because of the combination of strong thermal transport, substantial burnup capability, and favorable safety and cost attributes [3,4], dispersion fuels are well suited for research and test reactors as well as certain nuclear waste applications [5,6,7,8,9,10]. The harsh in-reactor environment drives the production of large quantities of solid and gaseous fission products within the particles, which, in turn, induces irradiation swelling and strongly modulates the mechanical response of dispersion fuel elements during service. Therefore, it follows that a clear grasp of irradiation effects is essential for assessing service life and for tuning the microstructural design to achieve reliable performance.

As a cornerstone of advanced nuclear energy systems, dispersion fuels have remained central to nuclear engineering research. From early theoretical treatments [1,11,12] through to later work on fission gas swelling, irradiation hardening, and creep [13], the field has advanced steadily. Many studies still lean on idealized assumptions or simplified abstractions that miss the intricate, evolving interactions between fuel particles and their surrounding matrix. With modern computational tools maturing rapidly, high-fidelity numerical simulation has become indispensable for interrogating dispersion fuel behavior in a more realistic manner.

Under irradiation, fuel elements experience tightly coupled thermal, mechanical, chemical, and radiation effects that trigger a suite of nonlinear processes [6,14]. Macroscale deformation governs the development of total strain, stress fields, and temperature distributions within the fuel body [15]. In a dispersed system, this deformation caused by expansion and creep due to irradiation is generated by the combined response of particles and the matrix they occupy [16]. The pronounced swelling of fuel particles drives strong mechanical interaction with the inert matrix. This interaction shapes stress-assisted creep while being simultaneously limited by the matrix’s creep resistance [17]. From this perspective, evaluating the thermomechanical response of dispersed fuels requires linking mesoscale physics with effective volume characteristics [18,19]. Although homogenized fuel behavior under irradiation has been widely examined [20,21], direct experimental data for true dispersion systems remain sparse. Considering the cost and duration of irradiation activities, numerical methods based on homogenization are often used to estimate equivalent properties [22,23]. For example, Dong et al. [22,24] computed the effective thermal conductivity of dispersion plates under in-pile heat transfer and paired those simulations with deep learning for rapid inference. However, their work is restricted to conductivity and does not generalize to other properties. Similarly, Gong et al. [25,26] used finite elements to evaluate particle swelling, matrix and cladding hardening, creep, and irradiation-induced growth. However, the expansion model relies on the empirical correlation of traditional rod-type fuels, and when the fissile gas is trapped in particles, this model often underestimates the expansion. Therefore, a new model combining the fissile gas mechanism is needed to capture the coupled thermomechanical response of dispersed fuels more precisely.

Commercial finite element platforms (including MATHEMATICA [27], ANSYS [28], and ABAQUS [29]) have been used to simulate the thermal and mechanical behaviors of nuclear fuel systems. Among these, ABAQUS has seen particularly broad use in studies of dispersed fuel plates [30], SiC/ZrC-coated TRISO fuel particles [31], fully ceramic microencapsulated fuel pellets [32], and dispersed TRISO-coated particle fuel plates [33].

In this study, we advanced a mesoscale numerical method grounded in composite micromechanics. Fuel particles are arranged in a simple cubic periodic architecture, and a representative volume element (RVE) is employed to resolve local stress–strain responses alongside the aggregate deformation. The formulation-coupled irradiation induced hardening of the metallic matrix with particle swelling described by a mechanism-based model tied to fission gas release. We used ABAQUS to examine how particle size, volume fraction, operating temperature, and burnup influence the effective thermomechanical properties of dispersion fuels. The results support the optimization and safety assessment of UO_2_–Zr dispersion fuel elements at the reactor scale.

## 2. Physical Model and Material Properties

A finite element model considering the geometry of the dispersed fuel and the symmetry of the load was established and simulated using the ABAQUS 2020 standard solver. Combining the physical properties and constitutive models of UO_2_ and Zr alloys, the material constitutive relationship and stress update algorithm are realized through user-defined subroutines.

### 2.1. Finite Element Modeling Methods

**Figure 2:**
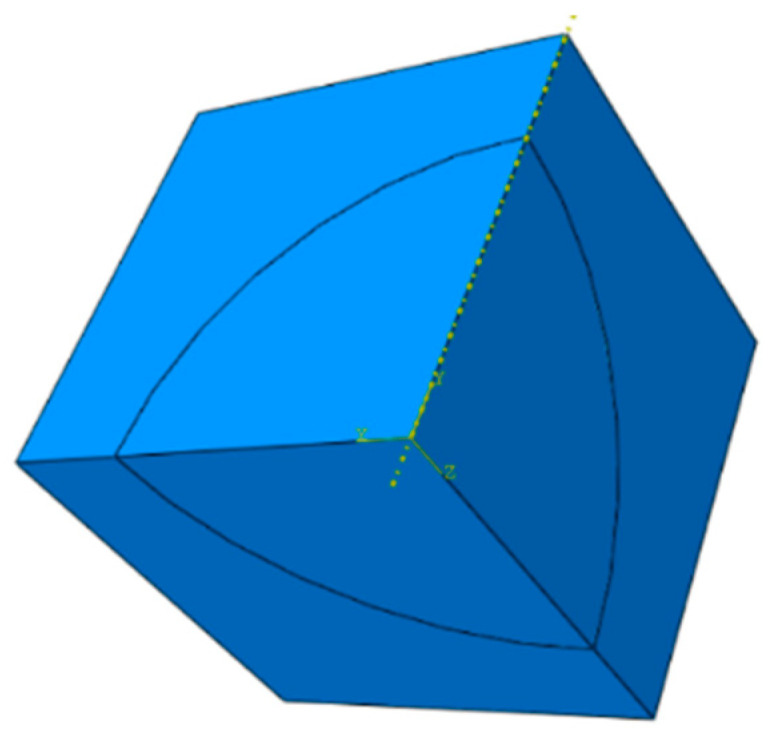
Finite element geometric model.

The structure of dispersed fuels resembles that of particle-reinforced composites. The RVE method is commonly employed to analyze composite material properties [34]. The RVE is embedded within an infinite UO_2_/Zr dispersion fuel rod, and by symmetry, its boundaries remain planar as fuel burnup increases. Exploiting both structural and loading symmetries, one-eighth of the RVE is chosen for a simplified finite element analysis. The corresponding geometric model is illustrated in Figure 2.

### 2.2. Finite Element Mesh

**Figure 3:**
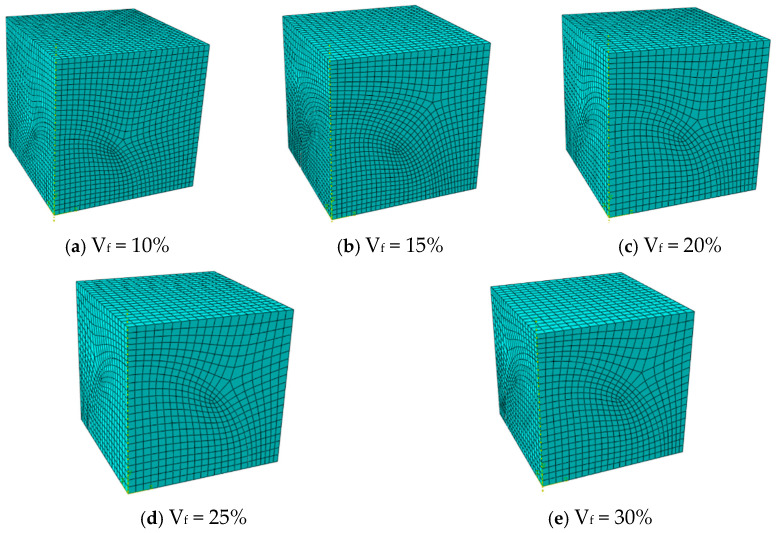
Finite element models for different particle volume contents.

**Figure 4:**
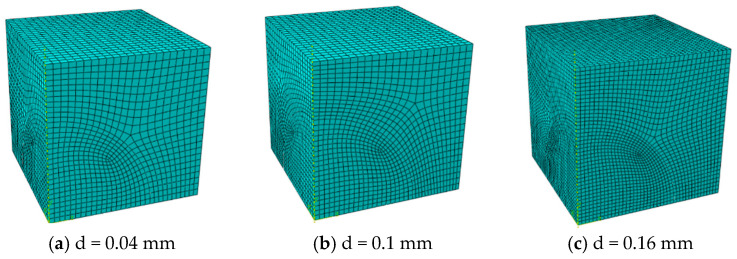
Finite element model of different particle diameters at 15% volume content.

**Figure 5:**
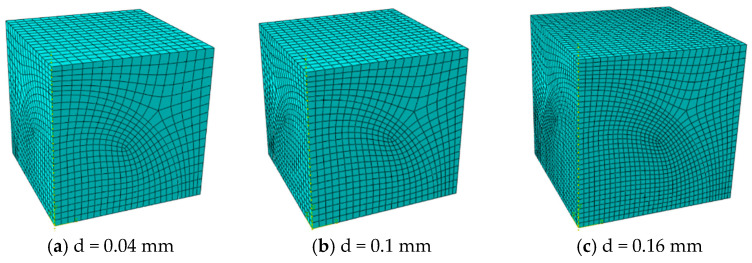
Finite element model of different particle diameters at 30% volume content.

The model is meshed using C3D8RT (three-dimensional 8-node thermally coupled hexahedral reduced integration) elements. Suppose spherical particles are arranged in a simple cubic pattern with a fixed diameter of 0.1 mm, and consider five particle volume fractions (10%, 15%, 20%, 25%, and 30%). Adjust the model size to obtain the corresponding volume fraction. The corresponding geometric model and mesh information are shown in Figure 3 and Table 1, respectively. In addition, when the volume fraction was fixed at 15% and 30%, the influence of particle size (with diameters of 0.04 mm, 0.1 mm, and 0.16 mm respectively) was studied. The corresponding finite element mesh data are shown in Table 2, and the model geometry is shown in Figure 4 and Figure 5.

### 2.3. Numerical Simulation Methods

To evaluate the equivalent properties of dispersion fuels, the burnup interval is partitioned into several incremental time steps. Due to the complex material properties and large deformation, a three-dimensional stress renewal scheme for fuel particles and the matrix was established in the rotating coordinate system.

For a typical incremental step $\left[t,t+\Delta t\right]$, the temperature at an integration point increases from T to T+ΔT. In the rotating coordinate system, the Cauchy stress $\sigma_{ij}^{t}$ at the beginning of the increment and the corresponding elastic strain $\varepsilon_{ij}^{e(t)}$ satisfy

(1) $$\sigma_{ij}^{t}=2G(T,t)\varepsilon_{ij}^{e(t)}+\lambda (t,T)\varepsilon_{kk}^{e(t)}\delta_{ij}$$

At the end of the incremental step, the Cauchy stress and the elastic logarithmic strain satisfy

(2) $$\sigma_{ij}^{t+\Delta t}=2G\left(T+\Delta T,t+\Delta t\right)\varepsilon_{ij}^{e(t+\Delta t)}+\lambda \left(t+\Delta t,T+\Delta T\right)\varepsilon_{kk}^{e(t+\Delta t)}\delta_{ij}$$

where $\sigma_{ij}^{t+\Delta t},\varepsilon_{ij}^{e(t+\Delta t)}$ represent the Cauchy stress at the end of the increment step corresponding to the logarithmic elastic strain, while $G\left(T+\Delta T,t+\Delta t\right)$ and $\lambda \left(t+\Delta t,T+\Delta T\right)$ denote the Lamé constants.

The stress increment within this increment step can be expressed as

(3) $$\Delta \sigma_{ij}=2G\left(t+\Delta t,T+\Delta T\right)\Delta \varepsilon_{ij}^{e}+\lambda \left(t+\Delta t,T+\Delta T\right)\Delta \varepsilon_{kk}^{e}\delta_{ij}+\Delta \sigma_{ij}^{0}$$

Here, $\Delta \sigma_{ij}^{0}=2\Delta G\varepsilon_{ij}^{e(t)}+\Delta \lambda \varepsilon_{kk}^{e(t)}\delta_{ij}$ is related to the elastic strain at the beginning of the increment step and is a known quantity.

For the matrix material, the irradiation induced hardening, plasticity, and creep effects are considered as follows:

(4) $$\Delta \varepsilon_{ij}^{e}=\Delta \varepsilon_{ij}^{total}-\Delta \varepsilon_{ij}^{cr}-\Delta \varepsilon_{ij}^{p}$$

In the equation, $\Delta \varepsilon_{ij}^{total}$ is the total strain increment, while $\Delta \varepsilon_{ij}^{cr}$ and $\Delta \varepsilon_{ij}^{p}$ denote the creep strain increment and the equivalent plastic strain increment, respectively.

(5) $$\Delta \varepsilon_{ij}^{cr}=\frac{3s_{ij}^{t+\Delta t}}{2{\bar{\sigma }}^{t+\Delta t}}\Delta {\bar{\varepsilon }}^{cr}({\bar{\sigma }}^{t+\Delta t},t+\Delta t,T+\Delta T)$$

(6) $$\Delta \varepsilon_{ij}^{P}=\frac{3s_{ij}^{t+\Delta t}}{2{\bar{\sigma }}^{t+\Delta t}}\Delta {\bar{\varepsilon }}^{p}$$

Assuming no additional plastic strain increment, the trial stress is calculated. According to the definition of the von Mises equivalent stress $\bar{\sigma }=\sqrt{3s_{ij}s_{ij}/2}$, the nonlinear equation satisfied by ${\bar{\sigma }}^{pr1}$ and $\Delta {\bar{\varepsilon }}_{cr}$ can be expressed as

(7) $${\bar{\sigma }}^{pr1}+3G\Delta {\bar{\varepsilon }}_{cr}\left({\bar{\sigma }}^{pr1},t+\Delta t,T+\Delta T\right)={\bar{\sigma }}^{pr2}$$

The nonlinear equation is solved by employing the Newton–Raphson iterative method. When the convergence criterion $\left|\frac{\delta {\bar{\sigma }}^{\left(k+1\right)}}{{\bar{\sigma }}^{\left(k+1\right)}}\right|\leq 1\times 10^{-6}$ is satisfied, the trial stress ${\bar{\sigma }}^{pr1}$ is obtained.

If ${\bar{\sigma }}^{pr1}<\left(1+1\times 10^{-6}\right)\sigma_{y}\left({\bar{\varepsilon_{p}}}^{t},t+\Delta t,T+\Delta T\right)$, no additional plastic strain increment occurs within the increment step. Here, $\sigma_{y}\left({\bar{\varepsilon_{p}}}^{t},t+\Delta t,T+\Delta T\right)$ is the yield stress calculated according to the irradiation hardening model, and ${\bar{\sigma }}^{pr1}$ denotes the true stress, which is then used to update the stress–strain state.

If ${\bar{\sigma }}^{pr1}>\left(1+1\times 10^{-6}\right)\sigma_{y}\left({\bar{\varepsilon_{p}}}^{t},t+\Delta t,T+\Delta T\right)$, a new plastic strain increment occurs within the increment step. Following a derivation similar to the case with no additional plastic strain increment, the nonlinear equation satisfied by ${\bar{\sigma }}^{t+\Delta t}$, $\Delta {\bar{\varepsilon }}_{p}$ and $\Delta {\bar{\varepsilon }}_{cr}$ can be expressed as

(8) $${\bar{\sigma }}^{t+\Delta t}+3G\Delta {\bar{\varepsilon }}_{cr}\left({\bar{\sigma }}^{t+\Delta t},t+\Delta t,T+\Delta T\right)+3G\Delta {\bar{\varepsilon }}_{p}={\bar{\sigma }}^{pr2}$$

Since $\left[\Delta {\bar{\varepsilon }}_{p}^{t+\Delta t},{\bar{\sigma }}^{t+\Delta t}\right]$ must lie on the yield surface, $\Delta {\bar{\varepsilon }}_{p}^{t+\Delta t}$ and ${\bar{\sigma }}^{t+\Delta t}$ satisfy the following condition:

(9) $${\bar{\sigma }}^{t+\Delta t}=\sigma_{y}\left({\bar{\varepsilon }}_{p}^{t}+\Delta {\bar{\varepsilon }}_{p},t+\Delta t,T+\Delta T\right)$$

Meanwhile, using the relationship between the creep strain increments $\Delta {\bar{\varepsilon }}_{cr}$ and ${\bar{\sigma }}^{pr1}$, an equation for the equivalent plastic strain increment is derived:

(10) $$g\left(\Delta {\bar{\varepsilon }}_{p}\right)={\bar{\sigma }}^{t+\Delta t}\left({\bar{\varepsilon }}_{p}^{t}+\Delta {\bar{\varepsilon }}_{p}\right)+3G\Delta {\bar{\varepsilon }}_{cr}\left({\bar{\sigma }}^{t+\Delta t}\left({\bar{\varepsilon }}_{p}^{t}+\Delta {\bar{\varepsilon }}_{p}\right)\right)3G\Delta {\bar{\varepsilon }}_{p}-{\bar{\sigma }}^{pr2}=0$$

The nonlinear equation is solved using the iterative method. When the convergence criterion $\left|\frac{\delta {\bar{\varepsilon }}_{p}^{\left(k+1\right)}}{{\bar{\varepsilon }}_{p}^{\left(k+1\right)}}\right|\leq 1\times 10^{-6}$ is satisfied, the equivalent plastic strain increment $\Delta {\bar{\varepsilon }}_{p}$ is obtained. The Mises stress at the end of the increment was determined using the plastic hardening function, and the stress–strain state was updated.

For the fuel particles, irradiation-induced swelling is taken into account, while the matrix material’s elastic properties and plastic behavior evolve over time. At each integration point within the fuel particles, the stress update procedure follows a method similar to that used for the matrix material.

### 2.4. Material Properties

UO_2_ ceramics have the advantages of high melting point, radiation resistance, low swelling rate, and strong retention of fission products, and are widely used in the design and manufacture of diffuse nuclear fuel elements. Considering the maturity and reliability of UO_2_–Zr alloy structures in existing nuclear reactors [33,34], UO_2_–Zr alloy materials were selected as the subject of this study.

The elastic modulus and Poisson’s ratio of UO_2_ fuel pellets are determined as follows [35]:

(11) E=162000+63000/(1+Bu)−20(T+273)

(12) v=0.316

where E is the elastic modulus of UO_2_ fuel particles; *Bu* is fuel consumption; T denotes the temperature; and υ is the Poisson’s ratio.

The thermal conductivity model of UO_2_ [36]:

(13) $$\begin{array}{l} k=\left(\frac{D}{1+\left(6.5-0.00469T^{\prime }\right)\left(1-D\right)}\right)\left(\frac{C_{V}}{(A+BT^{\prime \prime })\left(1+3e_{th}\right)}\right)\\ +5.2997\times 10^{-3}T\left[\exp\left(-\frac{13358}{T}\right)\right]\left\{1+0.169{\left[\left(\frac{13358}{T}\right)+2\right]}^{2}\right\} \end{array}$$

where k is the thermal conductivity of UO_2_; *D* is the theoretical density of the particles; $C_{V}$ denotes the effect of phonon specific heat at constant volume; $e_{th}$ is the thermal expansion coefficient when the temperature is above 300 K; $T^{\prime }$ and $T^{\prime \prime }$ denote correction factors of porosity with respect to temperature; A is the influence factor of point defects on phonon diffusion; and B is the influence factor of phonon–phonon scattering on phonon diffusion.

The thermal expansion strain of UO_2_ fuel particles is calculated as follows [36]:

(14) $$\frac{\Delta L}{L_{0}}=K_{1}T-K_{2}+K_{3}e^{-\frac{E_{D}}{kT}}$$

where $\frac{\Delta L}{L_{0}}$ is the linear thermal expansion; *E_D_* is the dislocation formation energy, with a value of $6.9\times 10^{-20}$ in this study; $K_{1}=1.0\times 10^{-5};K_{2}=3.0\times 10^{-3};K_{3}=4.0\times 10^{-2}$; and *k* is the Boltzmann constant.

The total volumetric change of fuel particles due to irradiation swelling comprises contributions from both solid and gaseous fission products.

(15) $$SW^{fuel}=SW^{ss}+SW^{gs}$$

The diffusion of fission gas within the grains is governed by the diffusion equation in one-dimensional spherical coordinates:

(16) $$\frac{\partial C_{t}}{\partial t}=D_{eff}\frac{1}{r^{2}}\frac{\partial }{\partial r}\left(r^{2}\frac{\partial C_{t}}{\partial r}\right)+\beta$$

where $C_{t}$ is the total number of intragranular gas atoms per unit volume; $D_{eff}$ is the effective diffusion coefficient; r denotes the radial coordinate of the spherical grain; and β is the generation rate of gas atoms. The calculation of $D_{eff}$ adopts the model in TRANSURANUS [37], in which intragranular bubbles are considered a special type of defect. On the one hand, they capture fission gas atoms. On the other hand, due to the intragranular resolution effect caused by fission fragment impacts, part of the fission gas atoms is re-dissolved back into the grain. Accordingly, the effective diffusion coefficient is calculated as

(17) $$D_{eff}=\frac{b}{g+b}D_{s}+\frac{g}{g+b}D_{b}$$

where $\frac{b}{g+b}$ is the fraction of fission gas atoms participating in diffusion as single gas atoms, and $\frac{g}{g+b}$ is the fraction of fission gas atoms inside bubbles that diffuse together with the bubbles.

The capture rate and re-dissolution rate are calculated as follows:

(18) $$g=4\pi D_{s}R_{ig}N_{ig}$$

(19) $$b=3\times 10^{-23}F$$

where $R_{ig}$ is the average radius of intra-granular bubbles;$N_{ig}$ is the number of bubbles per unit volume; and *F* is the fission rate.

The diffusion coefficient of bubbles can be expressed as

(20) $$D_{b}=\frac{3\Omega_{ig}}{4\pi R_{ig}^{3}}D_{v}$$

(21) $$D_{v}=3\times 10^{-5}\exp\left(-4.5/\left(k_{b}T\right)\right)$$

where $\Omega_{ig}$ is the volume occupied by a single fission gas atom in the bubble and $k_{b}$ is the Boltzmann constant.

Grain boundary bubbles grow by absorbing fission gas atoms and vacancies [15]. The growth rate of grain boundary bubbles can be expressed as

(22) $$\frac{dV_{gf}}{dt}=\omega \frac{dn_{g}}{dt}+\Omega_{gf}\frac{dn_{v}}{dt}$$

where $\frac{dn_{g}}{dt}$ is the absorption rate of fission gas atoms; ω is the van der Waals volume of fission gas atoms; $\frac{dn_{v}}{dt}$ is the absorption rate of vacancies by grain boundary bubbles; and Ω is the volume occupied by a single vacancy.

The rate at which grain boundary bubbles absorb vacancies can be expressed as

(23) $$\frac{dn_{v}}{dt}=\frac{2\pi D_{v}\delta_{g}}{k_{b}Ts}\left(p-p_{eq}\right)$$

where $n_{v}$ is the number of vacancies in a single bubble; $D_{v}$ is the diffusion coefficient of vacancies along the grain boundary; $\delta_{g}$ is the thickness of the grain boundary diffusion layer; $s=-\left(\left(3-F_{c}\right)\cdot \left(1-F_{c}\right)+2\ln\left(F_{c}\right)\right)/4$; $F_{c}$ is the area fraction of grain boundary bubbles on the grain boundary; p is the internal pressure of the grain boundary bubble; and $p_{eq}$ is the mechanical equilibrium pressure. For ellipsoidal bubbles with a circular projection, the mechanical equilibrium pressure is

(24) $$p_{eq}=\frac{2\gamma }{R_{gf}}+\sigma_{h}$$

where, γ is the surface energy; $R_{gf}$ is the curvature radius of the bubble; and $\sigma_{h}$ is the hydrostatic pressure, with a positive value indicating that the bubble is under compression.

For a van der Waals gas, the pressure inside the bubble satisfies

(25) $$p\left(V_{gf}-n_{g}\omega \right)=n_{g}k_{b}T$$

where $n_{g}$ is the average number of fission gas atoms contained in a bubble and $V_{gf}$ is the volume of the bubble. Assuming that the bubble contains $n_{g}$ fission gas atoms and $n_{v}$ vacancies, the volume of the bubble is

(26) $$V_{gf}=n_{g}\omega +n_{v}\omega_{gf}$$

Here, $V_{gf}$ is the volume of the grain boundary bubble; $n_{g}$ is the number of fission gas atoms in the grain boundary; ω is the van der Waals volume of a fission gas atom; $n_{v}$ is the number of vacancies in the grain boundary; and $\omega_{gf}$ is the atomic volume occupied by a single vacancy within the bubble.

From the bubble volume, the curvature radius can be expressed as

(27) $$R_{gf}={\left(\frac{3V_{gf}}{4\pi \varphi \left(\theta \right)}\right)}^{1/3}$$

Here, θ is the half-dihedral angle of the bubble: $\varphi (\theta )=1-1.5\cos\theta +0.5\cos^{2}\theta$.

The coalescence of grain boundary bubbles leads to bubble growth and a reduction in their total number along the grain boundary. Assuming that, during bubble coalescence, the total bubble volume per unit area remains conserved, the number of bubbles $N_{gf}$ per unit area decreases while the total bubble volume remains unchanged. Consequently, bubble growth results in an increase in the projected bubble area. The reduction rate of grain boundary bubbles resulting from coalescence can be expressed as

(28) $$\frac{dN_{gf}}{dt}=\frac{6N_{gf}^{2}}{3+4N_{gf}A_{gf}}\frac{dA_{gf}}{dt}$$

where $N_{gf}$ is the number of grain boundary bubbles per unit area, and $A_{gf}$ is the average projected area of grain boundary bubbles on the grain boundary.

The release of fission gas is determined based on the saturation level of grain boundary bubbles. Considering the combined effects of bubble coalescence and fission gas release, the relationship between the number of bubbles $N_{gf}$ per unit area on the grain boundary and the bubble projected area $A_{gf}$ is expressed as

(29) $$\left\{\begin{array}{l} \frac{dN_{gf}}{dt}=\frac{6N_{gf}^{2}}{3+4N_{gf}A_{gf}}\frac{dA_{gf}}{dt}\;F_{c}<F_{c,sat}\\ \frac{dN_{gf}}{dt}=-\frac{N_{gf}}{A_{gf}}\frac{dA_{gf}}{dt}\;\;\;\;F_{c}=F_{c,sat} \end{array}\right.$$

where $F_{c,sat}$ is the saturated projected area of the bubbles.

The swelling of grain boundary bubbles is expressed as

(30) $${\left(\frac{\Delta V}{V}\right)}_{gf}=\frac{1}{2}\frac{N_{gf}}{\left(1/3\right)r_{gf}}\left(\frac{4}{3}\pi R_{gf}^{3}\varphi \left(\theta \right)\right)$$

For solid UO_2_ fuel pellets, the post-irradiation volumetric change rate caused by solid fission products is calculated using the model in MATPRO [36]:

(31) $$S_{s}=2.5\times 10^{-29}B_{S}$$

Here, $B_{s}$ is the increase in burnup (fissions/m^3^) over a certain time period.

The thermal conductivity of the zirconium alloy is calculated using the following expression [36]:

(32) $$k=7.51+2.09\times 10^{-2}T-1.45\times 10^{-5}T^{2}+7.67\times 10^{-9}T^{3}$$

(33) $$E=\left(1.088\times 10^{11}-5.475\times 10^{7}T+K_{1}+K_{2}\right)/K_{3}$$

where E is the elastic modulus of the zirconium alloy; $K_{1}$ is the correction factor due to oxidation; $K_{2}$ is the correction factor due to cold working; and $K_{3}$ is the correction factor due to fast neutron fluence. The Poisson’s ratio of the zirconium alloy ν is taken as a constant 0.3.

The specific calculation formulas for each term are as follows:

(34) $$\begin{array}{l} K_{1}=\left(6.61\times 10^{11}+5.921\times 10^{8}T\right)\Delta \\ K_{2}=-2.6\times 10^{10}C\\ K_{3}=0.88+0.12\times \exp\left(-\frac{\phi }{10^{25}}\right) \end{array}$$

where C is cold working (the ratio in the dimensionless region) and ϕ is the fast neutron fluence.

The creep model of the zirconium alloy adopts the model in FEMAXI [38]:

(35) $$\overset{\cdot }{\varepsilon }=K\phi \left(\sigma +Be^{C\sigma }\right)\exp\left(-\frac{10000}{RT}\right)t^{-0.5}$$

where $K=5.129\times 10^{-29}$; $B=7.252\times 10^{2}$; $C=4.967\times 10^{-8}$; R=1.987; $\overset{\cdot }{\varepsilon }$ is the creep strain rate; σ is the stress; and t represents time.

The irradiation hardening plastic curve of zirconium alloy is calculated using the following model [39]:

(36) $$\sigma =K\varepsilon^{n}{\left(\frac{\overset{\cdot }{\varepsilon }}{10^{-3}}\right)}^{m}$$

where σ,ε are the true effective stress and true effective strain, respectively; $\overset{\cdot }{\varepsilon }$ is the true strain rate; *K* is the strength coefficient; *n* is the strain hardening coefficient; and m is the strain rate sensitivity exponent. The calculation formula of *K* in the above relation is expressed as follows:

When T≤ 730 K,

(37) $$K=1.0884\times 10^{9}-1.0571\times 10^{6}T$$

When 730 K <T< 900 K,

(38) $$\begin{array}{l} K=A_{1}+T\left[A_{2}+T\left(A_{3}+A_{4}T\right)\right]\\ A_{1}=-8.152540534\times 10^{9},A_{2}=3.368940331\times 10^{7}\\ A_{3}=-4.317334084\times 10^{4},A_{4}=1.769348499\times 10^{1} \end{array}$$

When 900 K ≤T,

(39) $$K=\exp\left(8.755+\frac{8663}{T}\right)$$

The strain hardening exponent n is calculated as follows:

When T< 850 K,

(40) $$n=-1.86\times 10^{-2}+T\left[7.110\times 10^{-4}-7.721\times 10^{-7}T\right]$$

When T≥ 850 K,

(41) n=0.027908

The strain rate sensitivity exponent *m* is calculated as follows:

When T≤ 730 K,

(42) m=0.02

When 730 K <T< 900 K,

(43) $$\begin{array}{l} m=A_{5}+T\left[A_{6}+T\left(A_{7}+A_{8}T\right)\right]\\ A_{5}=2.063172161\times 10^{1},A_{6}=-7.704552983\times 10^{-2}\\ A_{7}=9.504843067\times 10^{-5},A_{8}=-3.860960716\times 10^{-8} \end{array}$$

When 900 K <T< 1090 K,

(44) $$m=-6.47\times 10^{-2}+2.203\times 10^{-4}T$$

### 2.5. The Boundary Conditions

In a single fuel element, the number of fuel particles can be extremely large (on the order of 10^8^∼10^9^) and the reactor environment is both complex and extreme. These factors make it very difficult to directly and accurately calculate or simulate the thermomechanical behavior of the fuel elements. To overcome this challenge, dispersion nuclear fuel is often treated as an equivalent particle-reinforced composite. Micromechanical methods are employed to determine its effective thermomechanical properties. To determine the effective elastic properties and thermal expansion coefficient of metallic matrix dispersion fuel, appropriate boundary conditions are applied for each case, followed by the corresponding numerical calculations.

#### 2.5.1. Boundary Conditions of Equivalent Elastic Properties

When the fuel is subjected to a far-field uniform displacement boundary, periodic boundary conditions are applied on the basis of RVE to maintain the deformation compatibility between adjacent units. For simplification, these periodic conditions are approximated by the “plane remains plane” assumption [40]. At the particle–matrix interfaces, both displacement and temperature are enforced as continuous. Then, the effective elastic properties of the dispersed fuel are evaluated by uniaxial tensile simulation using the finite element model. The displacement boundary conditions are shown in Figure 2.

(1) Node displacement constraints are applied to the faces *x* = *L*, *y* = *L*, and *z* = *L* to ensure that these surfaces remain planar;

(2) Symmetric boundary conditions are applied on the surfaces *x* = 0, *y* = 0, and *z* = 0.

Based on the applied loading, the calculation is performed in two steps:

The first step is virtual irradiation. The initial redistribution of stress between the particles and the matrix is caused by particle expansion due to irradiation. The calculation in this step considers the influence of fission gas migration.

The second step involves a virtual numerical tensile test, in which a displacement load is applied along the *Z*-axis on the *z* = *L* face. This step is a mechanical field calculation without considering particle irradiation swelling or fission gas migration.

#### 2.5.2. Boundary Conditions of Equivalent Coefficient of Thermal Expansion

According to Figure 2, the boundary conditions for calculating the coefficient of thermal expansion are set as follows:

(1) Node displacement constraint equations are applied to the faces *x* = *L*, *y* = *L* and *z* = *L*, allowing free movement but ensuring that these surfaces remain plane;

(2) Symmetry boundary conditions are applied to the faces *x* = 0, *y* = 0, and *z* = 0;

(3) The model’s initial temperature is set to *T*.

### 2.6. Data Post-Processing

Based on these calculations, the macroscopic equivalent true stress and equivalent true strain are expressed as

(45) $$\sigma_{true}=\frac{rf_{z}}{\left(L+u_{x})\times (L+u_{y}\right)}$$

(46) $$\varepsilon_{true}=\frac{u_{z}}{L+u_{z}}$$

where $u_{x}$**,** $u_{y}$, and $u_{z}$ represent the displacements in each direction on the planes *x* = *L*, *y* = *L*, and *z* = *L*, respectively, while $rf_{z}$ denotes the resultant reaction force in the Z-direction on the plane *z* = *L*.

For the uniaxial compression test, the stress–strain relationship is expressed as follows:

(47) $$E=\frac{\sigma_{true}}{\varepsilon_{true}}$$

After applying a temperature rise to the representative volume element, the equivalent thermal expansion coefficient can be expressed as

(48) $$\alpha_{c}=\frac{\Delta L}{L}\frac{1}{\Delta T}$$

where $\alpha_{c}$ is equivalent thermal expansion coefficient, and ΔT is temperature rise.

At time *t*, the relationship between the true strain and engineering strain due to volumetric swelling is expressed by the following equation:

(49) $$\theta^{sw\left(t\right)}=\ln\left(1+\frac{\Delta V}{V}\left(t\right)\right)$$

where $\theta^{sw\left(t\right)}$ is the true strain due to volumetric swelling at time *t*, and $\Delta V/V\left(t\right)$ is the corresponding engineering strain.

## 3. Model Verification

As elastic performance, coefficient of thermal expansion, and irradiation expansion are the key factors affecting the evaluation of fuel performance, a verification study was conducted to verify the correct implementation of the physical model in ABAQUS. The calculation results are in good agreement with the theoretical values.

### 3.1. Verification of Elastic Constitutive Model

For the dispersion nuclear fuel with a particle volume fraction of 15%, the equivalent elastic properties were calculated without considering burnup and compared with the theoretical formulas. In mechanics, Eshelby’s equivalent eigenstrain principle is fundamental to homogenization theory in elasticity [41,42]. It replaces the heterogeneous material containing inclusions with a uniform medium by introducing intrinsic strain. Stress equivalence is ensured through the Eshelby tensor. This method has been extended to various inclusion morphologies [43,44,45]. Several theoretical models to estimate effective elastic properties are shown in Table 3.

**Figure 6:**
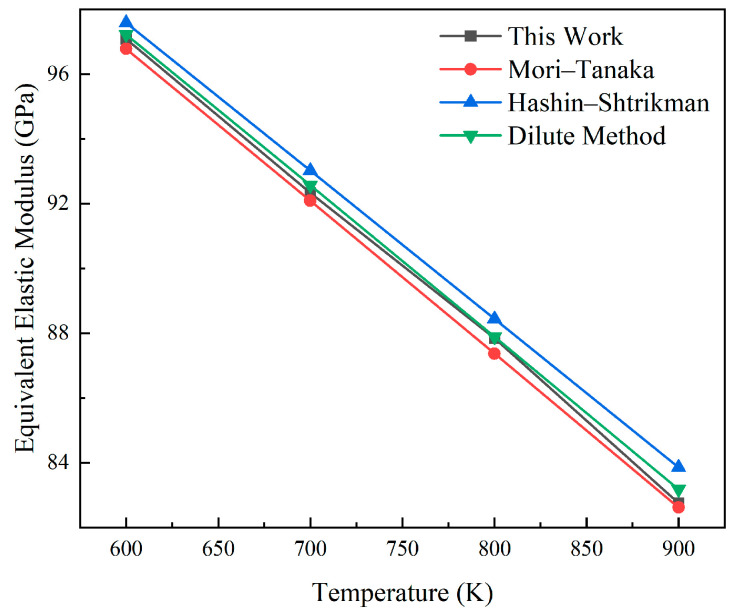
Verification plot of the equivalent elastic modulus.

**Figure 7:**
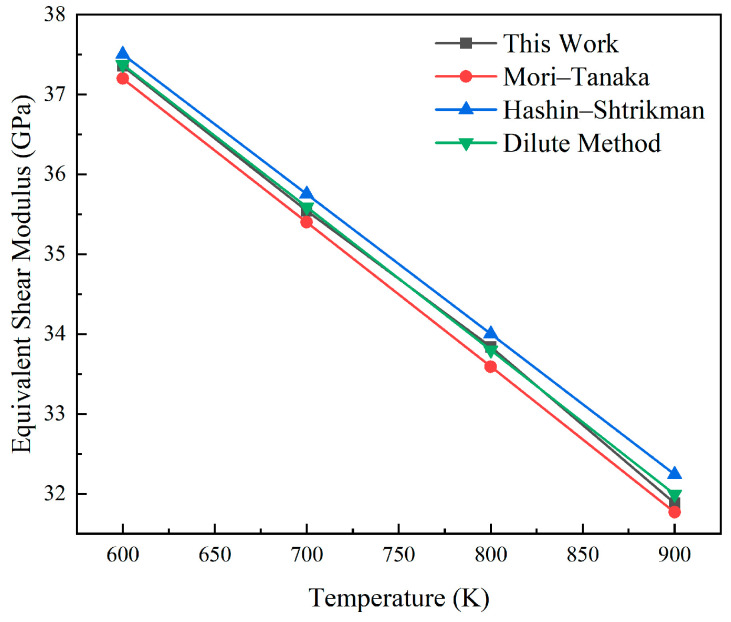
Verification plot of the equivalent shear modulus.

A cubic geometric specimen with a pellet volume fraction of 15% is constructed to simulate a uniaxial compression test. The theoretical elastic modulus at a given temperature *T* is calculated and compared with the value derived from finite element analysis. Figure 6 and Figure 7 show the comparison between the elastic modulus and shear modulus obtained from finite element calculations and theoretical solutions in the range of 600 K to 900 K. This method has good consistency with the established model. The errors of its equivalent elastic characteristics and shear modulus compared with the Mori–Tanaka model are less than 0.32% and 0.48%, respectively, and the errors compared with the Hashin–Shtrikman boundary are less than 0.81% and 0.64%, respectively. Compared with the method based on sparse solutions, the errors are, respectively, less than 0.25% and 0.11%. The high consistency between the calculation results and the theoretical values confirms the correctness of the calculations.

### 3.2. Verification of Thermal Expansion Model

The equivalent thermal expansion characteristics of dispersed nuclear fuel with a particle volume fraction of 15% were calculated without considering the fuel consumption and were compared with the theoretical prediction results. During the process of temperature change, the thermal expansion mismatch between fuel particles and the metal matrix will generate mechanical interactions at their interfaces. This strongly affects the overall thermal expansion behavior of the composite material. Several classical models to estimate the effective coefficient of thermal expansion are summarized in Table 4.

**Figure 8:**
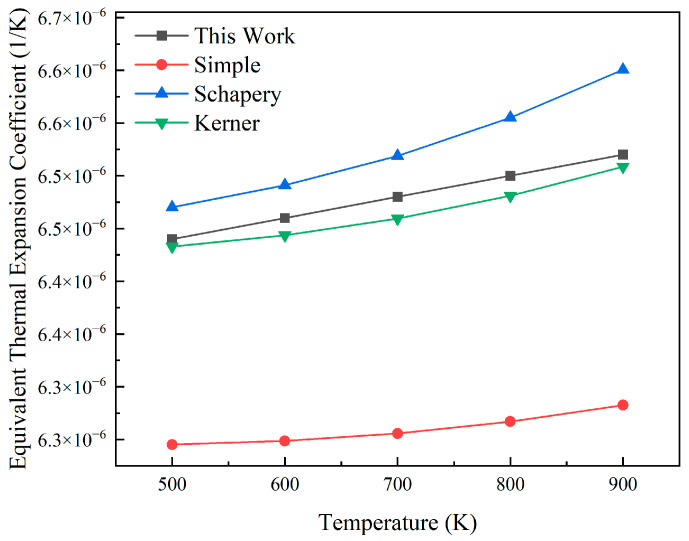
Verification plot of thermal expansion of the fuel.

Based on the fuel element geometry described in Section 2.1, a finite element model was established in ABAQUS. As shown in Figure 8, the equivalent coefficient of thermal expansion calculated when the volume fraction of fuel particles is 15% is compared with the theoretical model. The calculated coefficient of thermal expansion increases with temperature from 6.44 × 10^−6^ to 6.52 × 10^−6^. At a temperature of 500 K, the simple model error is 3.02%; at a temperature of 900 K, the simple model error is 3.64%. Due to the fact that the simple model does not take into account the interaction between particles and the matrix, it is found that the calculated equivalent coefficient of thermal expansion has a large deviation. Within the range of 500 K to 900 K, the calculated coefficients lie between the Schapery model and the Kerner model, and are closer to the Kerner model. Compared with the Kerner model, the error is less than 0.3%, and compared with the Schapery model, the error is less than 1.25%. The difference between the finite element results and the theoretical prediction is extremely small, indicating that the model simulates the thermal expansion behavior of the dispersed fuel well.

### 3.3. Verification of Irradiation Swelling of the Pellet

Irradiation swelling is a primary factor driving dimensional changes, so its behavior should be verified. At present, theoretical models for irradiation swelling remain incomplete. Most existing studies characterize and validate this phenomenon using empirical formulas [25]. The accuracy of the established irradiation expansion model is verified by comparing it with the empirical formula. Based on the particle irradiation expansion model in Section 2.4, the theoretical expansion of fuel pellets under different combustion quantities was calculated to obtain the corresponding volume true strain.

**Figure 9:**
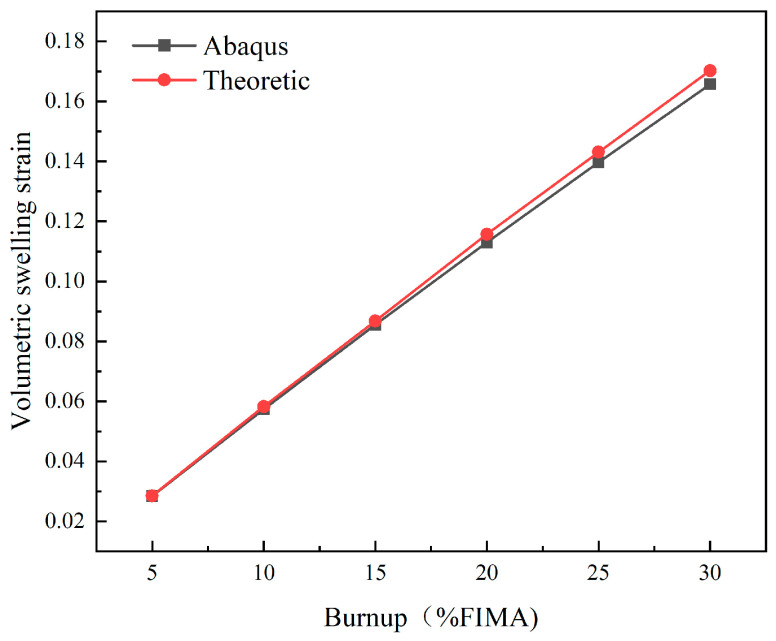
Verification plot of swelling strain of the pellet.

The total deformation of fuel particles includes elastic deformation, plastic deformation, and irradiation expansion. Among these factors, elastic deformation and irradiation swelling contribute to particle volumetric change. Since elastic compressive deformation is minor, irradiation swelling is the primary driver of expansion. Figure 9 shows the comparison between the theoretical and calculated values of radiation-induced swelling. The measured volume expansion is smaller than the theoretical expansion, and the difference between the two increases with the increase in fuel consumption. When the burnup is 5% FIMA, the error is less than 0.45%; when the burnup is 15% FIMA, the error is less than 1.4%; and when the burnup reaches 30% FIMA, the error reaches 2.5%. This is because the fuel particles are restricted by the surrounding metal matrix, generating elastic compressive strain within the particles. As fuel consumption increases, these pressures become more pronounced. The results show that ABAQUS can effectively simulate irradiation expansion when considering the release of fission gas.

## 4. Analysis of Numerical Results

In this section, the influences of pellet volume fraction, pellet size, temperature, and burnup on the equivalent elastic properties and thermal expansion coefficient of UO_2_–Zr dispersion nuclear fuel are calculated and analyzed.

### 4.1. Equivalent Elastic Model for Dispersion Fuels

**Figure 10:**
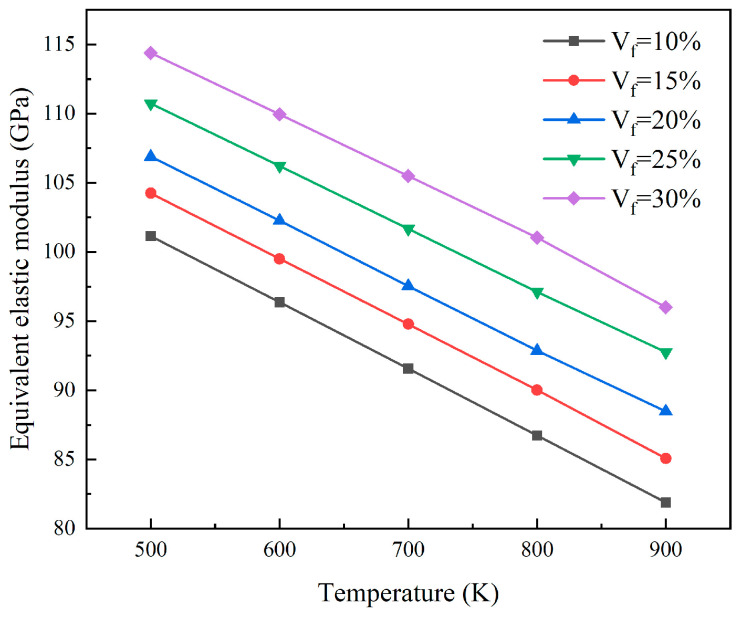
The plots of equivalent elastic modulus for different particle volumes.

**Figure 11:**
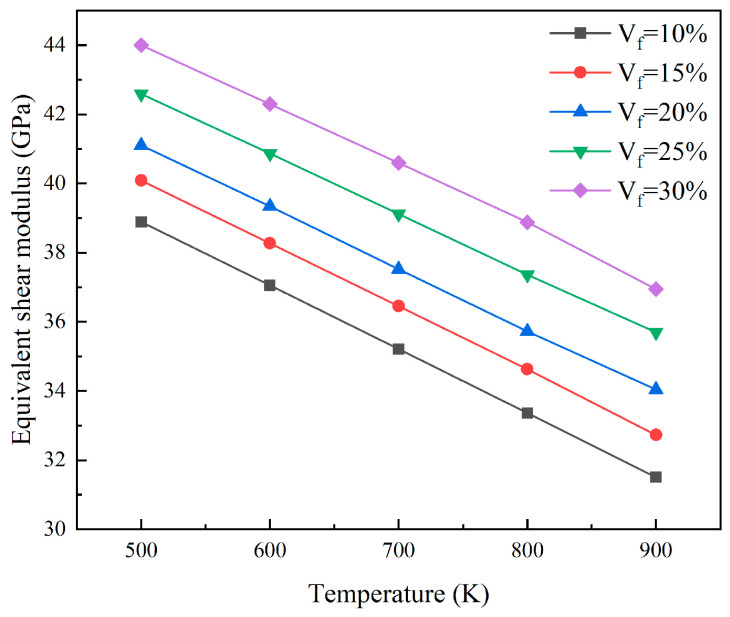
The plots of equivalent shear modulus for different particle volumes.

As shown in Figure 10, the equivalent elastic modulus of the reactor core was determined by analyzing the finite element models with different particle volume fractions at a 5% FIMA fuel consumption level. At the same temperature, the equivalent elastic modulus increases with particle volume fraction. For every 5% increase in particle volume fraction, the equivalent elastic modulus will increase by approximately 2.64 GPa to 4.28 GPa. As the temperature rises, the thermal motion of atoms intensifies, and the interatomic forces weaken, thereby reducing the overall stiffness of the material. Consequently, at a fixed particle volume fraction, the equivalent elastic modulus of the core exhibits a negative correlation with temperature. For every 100 K increase in temperature, the elastic modulus decreases by approximately 4.66 GPa. Figure 11 shows the equivalent shear modulus of the core at different particle volume fractions and temperatures under the same burnup level. The equivalent shear modulus shows a similar trend to the equivalent elastic modulus. At the same temperature, an increase in particle volume fraction leads to an increase in equivalent shear modulus. When the particle volume fraction increases by 5%, the equivalent shear modulus increases by approximately 1.01 GPa to 1.65 GPa. When the particle volume fraction remains constant, an increase in temperature will cause a decrease in the equivalent shear modulus. For every 100 K increase, the equivalent shear modulus decreases by approximately 1.79 GPa.

**Figure 12:**
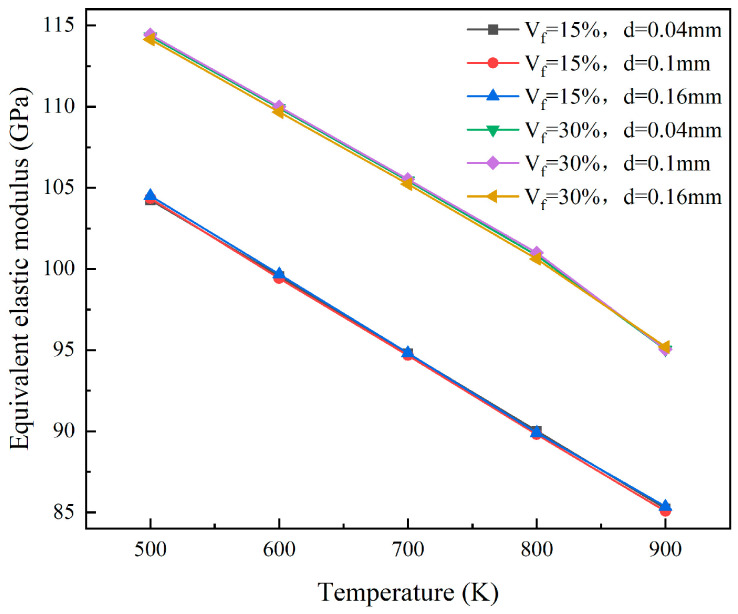
The plots of equivalent elastic modulus of different particle sizes.

**Figure 13:**
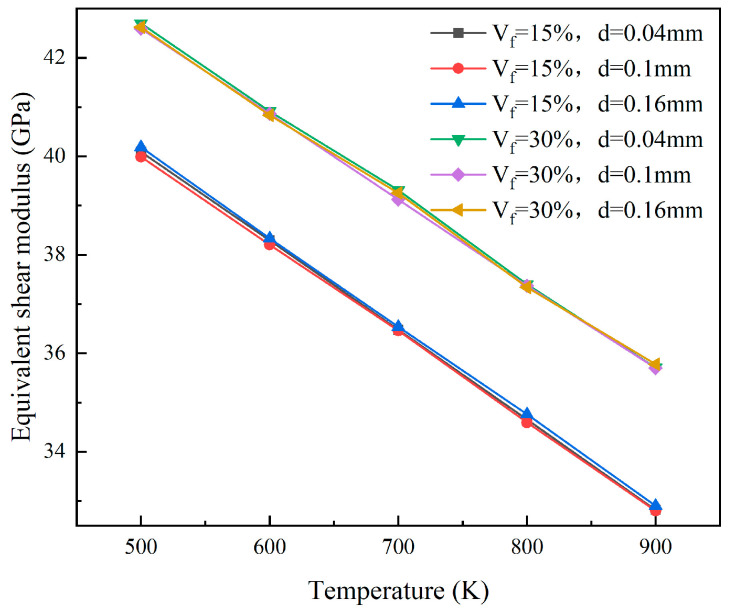
The plots of equivalent shear modulus of different particle sizes.

At the 5% FIMA fuel consumption level, consider the situation where the fuel particle radii are 0.02 mm, 0.05 mm, and 0.08 mm, respectively. The equivalent elastic properties of the models with particle volume fractions of 15% and 30% were calculated. The results for the equivalent elastic modulus and equivalent shear modulus are shown in Figure 12 and Figure 13, respectively. The variation in particle size has little influence on the equivalent elastic modulus and shear modulus. This is because, under the same fuel consumption, the mechanical interaction between the particles and the matrix changes very little, and the influence can be ignored.

**Figure 14:**
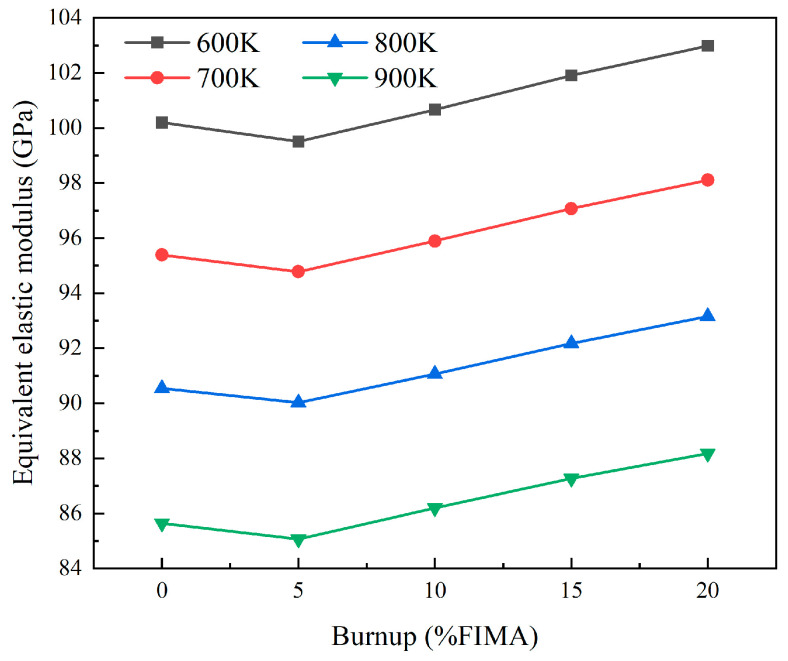
The plots of equivalent elastic modulus with different burnups.

**Figure 15:**
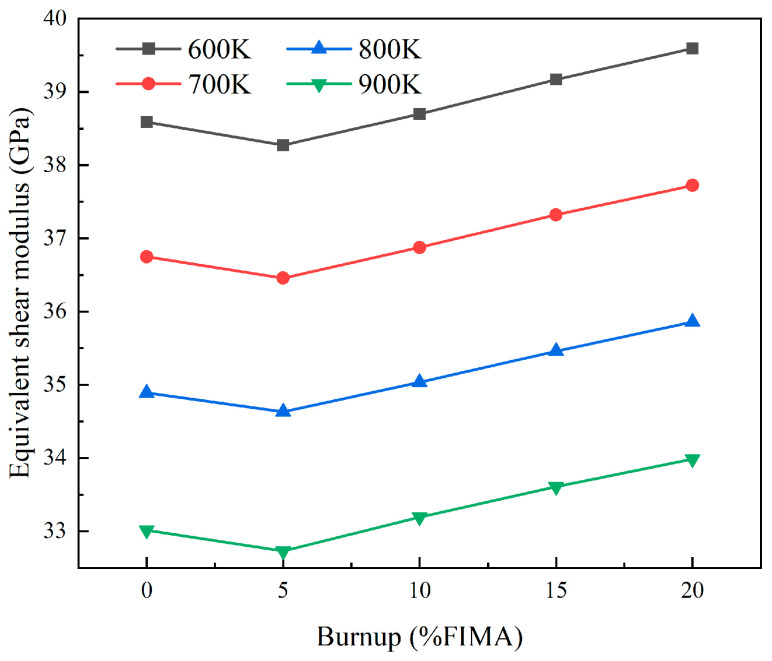
The plots of equivalent shear modulus with different burnups.

Figure 14 and Figure 15 show the relationship between burnup and the equivalent elastic and shear modulus for a model with a 25% particle volume fraction and 0.05 mm particle radius under different temperature conditions. At 900 K, the elastic modulus decreases to a minimum of 85.07 GPa at 5% FIMA, and then increases with increasing burnup, reaching 88.18 GPa at 20% FIMA. The trend of shear modulus and elastic modulus changes is the same, decreasing from 33.01 GPa to 32.73 GPa between 0% FIMA and 5% FIMA, and increasing to 33.98 GPa with increasing burnup.

With the increase in fuel consumption, both the equivalent elastic modulus and the shear modulus show a trend of first decreasing and then increasing. The elastic properties of fuel particles decrease as burnup increases [51]. In addition, irradiation can cause the volume of fuel particles to expand, which becomes more pronounced at higher combustion levels. At low fuel consumption, irradiation expansion is relatively small, and the decline in equivalent elastic performance is mainly caused by the reduction in particle elasticity. At higher fuel consumption, as the particle volume increases, both the elastic modulus and shear modulus of the particles are greater than those of the metal matrix. Fuel particles act as reinforcing phases within the reactor core, enhancing the equivalent elasticity of the reactor core.

### 4.2. Equivalent Coefficient of Thermal Expansion of Dispersion Fuels

**Figure 16:**
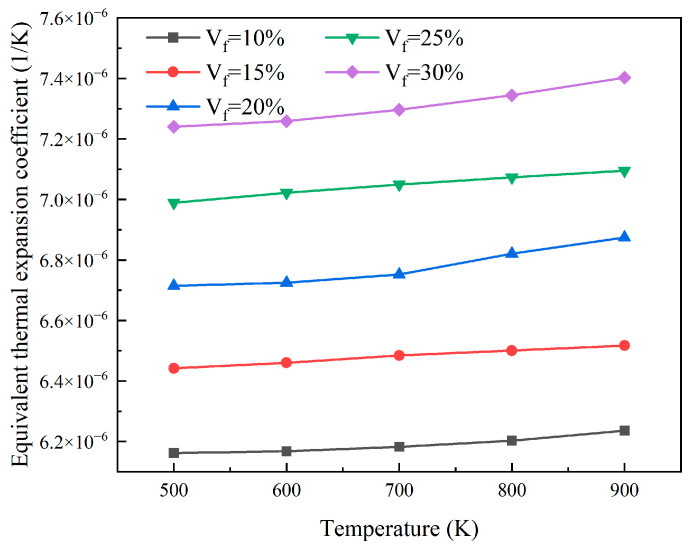
The plots of equivalent coefficient of thermal expansion at different particle volumes.

As shown in Figure 16, finite element calculations were performed for models $V_{f}=10\%$, $V_{f}=15\%$, $V_{f}=20\%$, $V_{f}=25\%$, and $V_{f}=30\%$. With the increase in the particle volume fraction, the coefficient of thermal expansion of the reactor core increases. This is because UO_2_ has a higher coefficient of thermal expansion than the surrounding matrix. With the increase in UO_2_ particle content, the total coefficient also increases accordingly.

**Figure 17:**
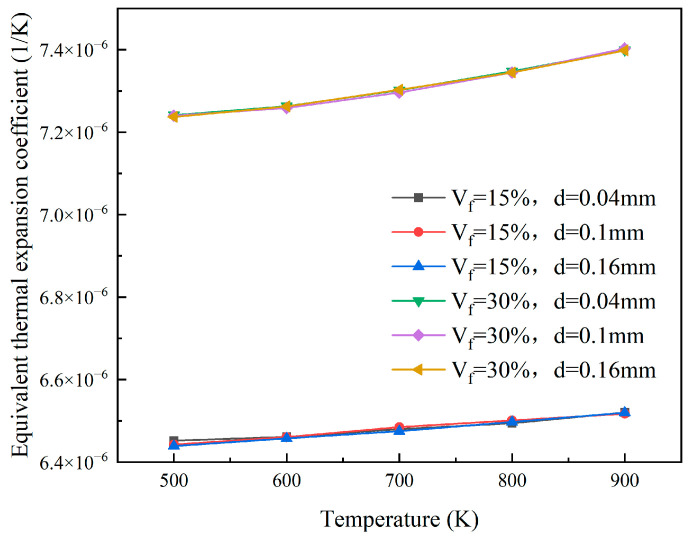
The plots of equivalent coefficient of thermal expansion of different particle sizes.

The finite element models with particle volume fractions of 15% and 30%, respectively, and particle radii of 0.02 mm, 0.05 mm, and 0.08 mm, respectively, were analyzed to obtain the equivalent thermal expansion coefficients under different temperature conditions. As shown in Figure 17, when the volume fraction of the particles is constant, the equivalent coefficient of thermal expansion is almost unaffected by the particle size. Under the condition of a fuel particle volume fraction of 30%, the equivalent thermal expansion coefficient increases from 7.24 × 10^−6^ at 500 K to 7.40 × 10^−6^ at 900 K. The equivalent thermal expansion coefficient of the reactor core increases with increasing temperature when the volume fraction of particles is constant.

## 5. Conclusions

In this study, we explicitly considered irradiation-induced matrix hardening and particle expansion and developed a theoretical approach to simulate the equivalent thermodynamic behavior of UO_2_-Zr rod-type dispersed fuel elements. This method combines thermal, mechanical, and fissile gas migration analysis. We evaluated the effects of fuel particle size, volume fraction, and fuel consumption on the equivalent thermodynamic performance of the reactor core. Within the range of the considered models and parameters, the main conclusions are as follows:

(1) Particle volume fraction, temperature, and burnup are the primary drivers of the core’s equivalent elastic properties. As the volume fraction of particles increases, the equivalent elastic modulus and shear modulus increase, while they decrease as the temperature rises. The fuel consumption shows a non-monotonic trend where the elastic modulus and shear modulus first decrease and then increase.

(2) Particle size has a negligible effect on the equivalent coefficient of thermal expansion, while particle volume fraction and temperature exert strong influences. Due to the high coefficient of thermal expansion of UO_2_, the equivalent thermal expansion of the composite material increases with the increase in particle volume fraction and further increases with the rise in temperature.

(3) The equivalent property parameters obtained in this study provide a foundation for optimizing dispersion fuel design.

## Figures and Tables

**Table 1:** Number of mesh nodes and elements with different particle volume contents.

Volume Fraction	Elements	Nodes
10%	21,755	24,795
15%	20,604	23,584
20%	15,896	18,468
25%	13,183	15,449
30%	10,072	12,104

**Table 2:** Number of mesh nodes and elements with different particle sizes.

Fuel Particle Diameter (mm)	Volume Fraction	Elements	Nodes
0.04	15%	15,872	18,362
0.1		20,604	23,584
0.16		48,224	53,496
0.04	30%	8465	10,153
0.1		10,072	12,104
0.16		24,697	28,179

**Table 3:** Equivalent elastic property models.

Method	Formula	Variable Description
Dilute method [46]	$K_{eff}=K_{m}\left(1+\frac{V_{f}\left(K_{f}/K_{m}-1\right)}{1+\left[\left(1+\nu_{m}\right)/3\left(1-\nu_{m}\right)-V_{f}\right]\left(K_{f}/K_{m}-1\right)}\right)$ $G_{eff}=G_{m}\left(1+\frac{V_{f}\left(G_{f}/G_{m}-1\right)}{1+\left[2\left(4-5\nu_{m}\right)/15\left(1-\nu_{m}\right)-V_{f}\right]\left(G_{f}/G_{m}-1\right)}\right)$	$K_{eff}$—Equivalent bulk modulus; $G_{eff}$—Equivalent shear modulus; $K_{f}$—Particle bulk modulus; $G_{f}$—Particle shear modulus; $\nu_{f}$—Particle Poisson’s ratio; $V_{f}$—Particle volume percentage; $K_{m}$—Matrix bulk modulus; $G_{m}$—Matrix shear modulus; $V_{m}$—Matrix volume percentage; $\nu_{m}$—Matrix Poisson’s ratio
Mori–Tanaka model [47,48,49]	$K_{eff}=K_{m}\left[1+\frac{V_{f}\left(K_{f}/K_{m}-1\right)}{1+\alpha \left(1-V_{f}\right)\left(K_{f}/K_{m}-1\right)}\right]$ $G_{eff}=G_{m}\left[1+\frac{V_{f}\left(G_{f}/G_{m}-1\right)}{1+\beta \left(1-V_{f}\right)\left(G_{f}/G_{m}-1\right)}\right]$ $\alpha =\frac{3K_{m}}{3K_{m}+4G_{m}}$ $\beta =\frac{6}{5}\cdot \frac{K_{m}+2G_{m}}{3K_{m}+4G_{m}}$	
Hashin–Shtrikman model [50]	$K_{eff}=K_{f}+\frac{V_{m}}{1/\left(K_{m}-K_{f}\right)+3V_{f}/\left(3K_{f}+4G_{f}\right)}$ $G_{eff}=G_{m}+\frac{V_{f}}{1/\left(G_{f}-G_{m}\right)+6V_{m}\left(K_{m}+2G_{m}\right)/5G_{m}\left(3K_{m}+4G_{m}\right)}$	

**Table 4:** Equivalent thermal expansion coefficient model.

Method	Formula	Variable Description
Simple model	$\alpha_{eff}=\alpha_{f}V_{f}+\alpha_{m}V_{m}$	$\alpha_{eff}$—Equivalent thermal expansion coefficient; $\alpha_{f}$—Particle thermal expansion coefficient; $\alpha_{m}$—Matrix thermal expansion coefficient; $K_{c}$—Bulk modulus calculated by Hashin–Shtrikman model
Kerner model	$\alpha_{eff}=\alpha_{f}V_{f}+\alpha_{m}V_{m}+(\alpha_{f}-\alpha_{m})V_{f}V_{m}\frac{K_{f}-K_{m}}{V_{f}K_{f}+V_{m}K_{m}+3K_{f}K_{m}/4G_{m}}$	
Schapery model	$\alpha_{eff}=\alpha_{f}+(\alpha_{m}-\alpha_{f})\frac{1/K_{c}-1/K_{f}}{1/K_{m}-1/K_{f}}$	

## Data Availability

The original contributions presented in the study are included in the article; further inquiries can be directed to the corresponding author.
